# Trends and correlates of girl-child marriage in 11 West African countries: evidence from recent Demographic and Health Surveys

**DOI:** 10.12688/aasopenres.13248.1

**Published:** 2021-06-22

**Authors:** Adesegun O. Fatusi, Sunday A. Adedini, Jacob Wale Mobolaji

**Affiliations:** 1 Department of Community Health, College of Health Sciences, Obafemi Awolowo University, Ile-Ife, Osun, 220005, Nigeria; 2 Academy for Health Development, AHEAD, Ile-Ife, Osun, 220005, Nigeria; 3 University of Medical Sciences, UNIMED, Ondo, Ondo State, Nigeria; 4 Demography and Social Statistics Department, Federal University Oye-Ekiti, Oye-Ekiti, Ekiti State, 371106, Nigeria; 5 Programme in Demography and Population Studies, Schools of Public Health and Social Sciences, University of the Witwatersrand, Johannesburg, 2050, South Africa; 6 Department of Demography and Social Statistics, Obafemi Awolowo University, Ile-Ife, Osun, 220005, Nigeria

**Keywords:** Child Marriage, Girl-Child Marriage, Early Marriage, West Africa

## Abstract

**Background**: West Africa historically has a high prevalence of girl-child marriage and requires substantial reduction to meet the United Nation’s Sustainable Development Goals (SDG) target of ending child marriage by 2030, but current data on progress is sparce. We aimed to determine the trend in child marriage in West Africa and assess the influence of selected socio-demographic factors.

**Methods**: We analysed data on women aged 18-24 years from the two most recent Demographic and Health Surveys (conducted between 2006 and 2014) for 11 West African countries to determine the prevalence and trend of girl-child marriage. Multivariable logistic regression analysis was used to assess the relationship between girl-child marriage and selected socio-demographic factors.

**Results**: The prevalence of child marriage in West Africa is 41.5%. An overall decrease of 4.6% (annual rate of 0.01%) was recorded over a seven-year inter-survey period. Three countries (Cote d’Ivoire, Nigeria, and Niger) recorded increased prevalence while the rate was unchanged in Burkina Faso, and the other six countries had reduced prevalence between the last two surveys. Sierra Leone recorded the highest decrease in prevalence (22%) and an annual reduction rate of 0.04%; Cote d’Ivore had the highest increase (65.3%). In virtually all countries, rural residence, low education, poor household economic status and non-Christian religious affiliation were significantly associated with higher odds of girl-child marriage.

**Conclusions**: The prevalence of girl-child marriage remains high in West Africa and the trend shows very slow progress. While substantial inter-country variations exist in overall rate and trend of child marriage, the rate of progress is inadequate across all countries.

## Introduction

Girl-child marriage, defined as marriage before the age of 18 years, is a global public health concern. It violates the human rights of the affected girls and increases their risks for several negative sexual and reproductive health outcomes, including maternal morbidity and mortality ^1– 3^, intimate partner violence ^2, 3^ and sexually transmitted infections ^2, 4^. Increased rates of adverse mental health conditions such as depression and suicidality have also been associated with girl-child marriage ^5, 6^. Girl-child marriage disrupts the schooling of the girl-bride and negatively impacts her development trajectory ^7^. Furthermore, children of girl-brides have an increased risk of childhood mortality and stunting ^8, 9^ as well as lower developmental opportunities and are more likely to be locked in an inter-generational cycle of poverty ^6^. In addition, girl-child marriage negatively affects national economies, with an estimated 9% reduction in adulthood earnings, mainly through its impact on education ^10^. The global aspiration to end girl-child marriage by 2030 is reflected in target 5.3 of the United Nation’s Sustainable Development Goals (SDGs) ^11^.

Whereas the proportion of girls aged 20–24 years who married before age 18 has slightly reduced from 25% to 21% over the last decade, the burden of child marriage remains quite high with an estimated 14.2 million girls affected annually ^11^. Globally, an estimated 650 million women currently alive have been child-brides ^9^. Sub-Saharan Africa contributes 18% of the global burden of girl-child marriage – the second highest after South Asia ^11^. However, with the significant progress in South Asia, which contrasts sharply with the slow progress in sub-Saharan Africa, the global burden of child marriage is shifting more to sub-Saharan Africa ^11^. Yet, as Petroni and colleagues recently pointed out, “far less attention has been paid to child marriage in sub-Saharan Africa” in relation to the high burden ^12^. In sub-Saharan Africa, the West and Central Africa sub-regions have the highest rates of girl-child marriage, with 41% of women aged 20–24 years married before 18 years in 2018 ^11^. Of the 10 countries with the highest rates of child-girl marriage in the world, four are in West Africa: Niger, Mali, Guinea, and Burkina Faso ^7, 13^.

Child marriage is an age-long practice influenced by poverty, low education, inequitable gender norms and low social status of women, among others ^7, 14^. Several events with implications – both positive and negative – for child marriage and its determinants have occurred in recent years globally, regionally, and nationally, and the rate of girl-child marriage may, therefore, be changing. The trend in child marriage and rate in the reduction of the prevalence may likely differ across countries, given the differences in political, social development, and legal contexts. For example, an increase in childhood education and urbanization has occurred at different rates in West African countries in recent years, with the potentials for reduced child-marriage prevalence. On the other hand, countries have also experienced economic challenges and conflicts, which may increase the risk of child-marriage ^15^. Furthermore, differential implementation of regional initiatives such as the Protocol to the African Charter on Human and Peoples’ Rights on the Rights of Women (Maputo Protocol) and African Union’s campaign launched in 2014 to end child marriage would likely affect the trend of child-marriage ^16^.

As an expert group convened by the World Health Organization (WHO) on research priorities noted, the “understanding of intra- and inter-country differences in child marriage is limited and more segmented analyses are needed that cover not only geographic variety but religion, ethnicity, education, social class” ^17^. Overall, the degree to which recent changes in the economic, politico-legal and social development landscapes have influenced the prevalence and determinants of child marriage in different West African countries is uncertain and the literature on this is sparse. Current data on trends in child marriage is also important for monitoring progress regarding SDG target 5.3. The primary objective of this study is to determine the prevalence and compare trends of girl-child marriage in West African countries. In addition, we assess the association between girl-child marriage and selected socio-demographic factors in the focal countries.

## Methods

### Data source

This study entailed a secondary analysis of the Demographic and Health Surveys (DHS) data on women aged 18–24 years in various West African countries. DHS is a nationally representative survey of key demographic and health indicators of men and women of childbearing ages (15 – 49 years), with interviewees selected using a two-stage stratified cluster sampling design ^18^. The clusters serve as the primary sampling units and are derived from the enumeration areas previously designed for the most recent population and housing census of each country. The DHS reports for each country detail the sampling design and data collection methods. We used the last two DHS rounds for each country to assess trends in child marriage, and the most recent survey to determine the current prevalence and correlates. The questions relating to the variables used in the analysis were similarly framed across all the DHS dataset used.

To be included in the study, a country must have had two DHS rounds undertaken within a 5- to 10-year time interval – a period long enough for noticeable changes to take place in the rate of child marriage. Eleven of the 16 West African countries met this criterion. We used the following DHS data for the countries: Benin – 2006 and 2012; Burkina Faso – 2003 and 2010; Cote d’Ivoire – 2005 and 2012; Ghana – 2008 and 2014; Guinea – 2005 and 2012; Liberia – 2007 and 2013; Mali – 2006 and 2012; Niger – 2006 and 2012; Nigeria – 2008 and 2013; Senegal – 2005 and 2014; and, Sierra Leone – 2008 and 2013.

### Variables measurements

Our outcome variable was child marriage, which was determined based on the respondents’ self-reported marital status and age at first marriage. The child marriage rate was the proportion of women aged 18–24 years who were married or in a union before age 18. For marital status, interviewees were asked the question, “what is your current marital status?” with the response options as “never in union”, “currently in union/living with a man”, and “formerly in union/formerly living with a man”. For age at marriage (for respondents in marriage or union), interviewees were asked: “How old were you when you first started living with husband/partner?” and a numeric response (in years) recorded.

The explanatory variables were included in the analysis based on extant literature. These were: (i) rural/urban place of residence; (ii) wealth quintiles used standardly in DHS as a measure of economic level and based on possession of some specified material things and type of housing (respondents were categorized into poorest, poorer, middle, richer and richest groups but re-categorised in some of our analyses as poorest/poorer, middle and richer/richest); (iii) religious affiliation (categorized into three initially – Christian, Muslim and others – but collapsed in the later analyses to Christian and non-Christians as the proportion of other religions was less than 5% for most countries; and, (iv) educational attainment (categorized into three groups – no formal education, primary, and secondary or higher education).

### Statistical analysis

Univariate analysis involving frequency and percentage distribution was used to determine the prevalence of girl-child marriage at two different time points for each country. Chi-square test of independence and multivariable logistic regression were used to statistically examine the associations between the outcome and the explanatory variables in the most recent DHS for each country. Recognizing that the educational level attained can be an outcome as well as a determinant of girl-child marriage, we ran two regression models – one with and the other without education. Generally, the age at primary school enrolment in West Africa is around 6 years while age at completion is around 12 years. Considering that just less than 3% of our sample had experienced marriage/union before age 12 (result not shown), lack of education is therefore unlikely to be an outcome of child marriage as the proportion with the possibility of dropping out of primary school because of child marriage was a negligible part of our sample. All analyses were undertaken using Stata software version 15.1.

## Results

### Socio-demographic characteristics

As Table 1 shows, young females aged 18–24 years constituted approximately 70% of the females sampled from the dataset. There are substantial inter-country differences in the socio-demographic characteristics of females age 18–24 in terms of residence, education, wealth quintile, and religion at each survey point as well as in terms of changes between the two different survey points. For example, the proportion of respondents who were rural-dwellers ranged from 45.3% in Benin to 77.6% in Niger in the first survey, and from 34.8% in Liberia to 79.3% in Niger in the second (most recent) survey, with an overall average of 59.9% in the first survey and 56.9% in the second survey. Whereas Benin, Sierra Leone, and Niger recorded an increase in the proportion of rural dwellers between the first and second surveys, the other eight countries recorded a decrease. While Nigeria recorded an increase in the proportion of 18–24 years who had no education between the two survey points (from 29.3% to 32.4%), all the other 10 countries recorded different levels of decrease. In addition, whereas Ghana, Nigeria, Mali, and Niger recorded an increase in the proportion of respondents who are in the poorer/poorest category, the other seven countries recorded varying levels of decrease. Overall, between the first and second survey, there was a reduction in the proportion of respondents who were rural-based (from 59.9% to 56.9%), had no education (from 18.9% to 17.0%) and were Christians (36.9% to 35.3%), while the economic distribution essentially remained the same.

**Table 1.** Percentage distribution of selected socio-demographic characteristics of young women (aged 18–24 years) in 11 West African countries.

Countries	Survey Year	Age	Percentage distribution among women aged 20–24 years									
			Residence	Education			Wealth quintile			Religion		
		% aged 20–24	% Rural	None	Primary	Secondary/ Higher	Poorer/ poorest	Middle	Richer/ richest	Christian	Muslim	Others ^a^
Ghana	2008 (n=1,298)	67.6	48.9	12.7	18.6	68.7	31.4	21.3	47.3	78.8	15.8	5.4
	2014 (n=2,227)	72.4	47.3	10.1	14.6	75.3	33.0	22.7	44.3	81.1	15.0	3.9
Benin	2006 (n=4,416)	72.0	45.3	54.4	21.6	24.0	31.5	19.0	49.5	59.4	22.1	18.5
	2012 (n=3,943)	73.4	49.9	46.0	18.8	35.2	30.4	17.7	51.9	61.9	20.9	17.2
Cote D’Ivoire	2005 (n=2,990)	69.1	49.9	41.2	25.6	33.2	31.9	19.4	48.7	46.6	34.4	19.0
	2012 (n=2,828)	69.1	43.9	46.3	26.1	27.6	31.3	19.2	49.5	45.2	41.2	13.6
Senegal	2005 (n=4,181)	68.0	49.8	54.7	29.2	16.1	33.7	19.0	47.3	4.3	95.7	0.0
	2014 (n=2,399)	69.1	44.8	39.0	21.9	39.1	33.3	20.1	46.6	4.0	95.5	0.5
Liberia	2007 (n=1,896)	71.9	53.1	27.1	40.1	32.8	33.3	19.2	47.5	87.7	9.0	3.2
	2013 (n=2,411)	68.1	34.8	17.8	33.2	49.0	31.2	19.1	49.7	85.7	10.6	3.7
Nigeria	2008 (n=8,730)	70.3	63.7	29.3	143	56.4	35.2	19.5	45.3	55.6	43.3	1.1
	2013 (n=9,709)	69.6	58.5	32.4	12.3	55.3	36.3	21.0	42.7	46.8	52.6	0.6
Sierra Leone	2008 (n=1,756)	67.6	57.1	53.6	15.3	31.1	32.7	17.7	49.4	22.4	76.0	1.6
	2013 (n=4,242)	63.3	57.3	34.5	15.2	50.3	31.6	17.4	51.0	24.7	74.6	0.7
Burkina Faso	2003 (n=3,313)	68.6	73.1	72.4	14.0	13.6	33.5	22.3	44.2	27.9	63.0	9.1
	2010 (n=4,589)	72.1	68.3	65.8	16.7	17.5	31.8	18.8	49.4	30.6	63.3	6.1
Guinea	2005 (n=1,740)	66.2	60.6	68.8	12.3	18.9	34.4	16.5	49.1	13.6	82.3	4.1
	2012 (n=2,505)	65.4	56.6	51.7	16.6	31.7	33.4	16.3	50.3	10.3	85.5	4.1
Mali	2006 (n=3,814)	70.2	61.2	72.7	11.8	15.5	32.0	18.3	49.7	3.3	91.9	4.8
	2012 (n=2,647)	69.7	71.5	64.4	11.6	24.0	34.7	17.1	48.2	3.9	93.1	3.0
Niger	2006 (n=2,306)	71.6	77.6	79.6	12.5	7.9	34.1	18.6	45.3	1.0	97.8	1.2
	2012 (n=2,698)	73.8	79.3	73.6	14.1	12.3	34.9	19.4	45.7	Nil	Nil	Nil
West Africa	Survey 1 (N=36,440)	69.7	59.9	50.2	18.9	30.9	33.4	19.3	47.4	36.9	57.0	6.1
	Survey 2 (N=40,201)	69.6	56.9	43.1	17.0	39.9	33.3	19.2	47.5	35.3	54.8	9.9

^a^Traditionalists, animists, no religion

### Trends in child marriage

Prevalence of child marriage ranged from 19.6% (in Cote d’Ivoire) to 74.1% (in Niger) in the first survey, and from 18.7% (in Ghana) to 76.3 (in Niger) in the second survey ( Table 2). Only Cote d’Ivoire (19.6%) and Ghana (21.6%) recorded a prevalence of less than 25% in the first survey, and only Ghana (18.7%) in the second survey. Whereas three countries recorded an increase in girl-child marriage prevalence – Cote d’Ivoire (65.3% increase), Nigeria (7.0% increase) and Niger (3.0% increase), the rate was virtually unchanged for Burkina Faso but the other seven countries recorded a decrease. The highest decrease was in Sierra Leone (22.0% reduction) over a 5-year period. The annual rate of change was minimal across the countries: Cote d’Ivoire (0.09%) recorded the highest annual rate of increase and Burkina Faso the highest annual rate of decrease (0.04%). Overall, a 4.6% decrease was recorded for the West African countries between the two survey points (from 43.5% to 41.5%) and an annual rate of 0.01% decrease.

**Table 2.** Trends in the prevalence of girl-child marriage in 11 West African countries.

Countries	Surveys	Prevalence of child marriage	Annual rate of change in prevalence	Total
**Country**				
Ghana	DHS 2008	21.6		1,298
	DHS 2014	18.7		2,227
	% change (95% CI)	-13.4 (11.6 – 15.4)	-0.022 (0.013 – 0.029)	
Benin	DHS 2006	33.4		4,416
	DHS 2012	29.5		3,943
	% change (95% CI)	-11.7 (10.8 – 12.7)	-0.019 (0.015 – 0.023)	
Cote d’Ivoire	DHS 2005	19.6		2,990
	DHS 2012	32.4		2,828
	% change (95% CI)	65.3 (63.5 – 67.0)	0.093 (0.083 – 0.104)	
Senegal	DHS 2005	38.1		4,181
	DHS 2014	31.3		2,402
	% change (95% CI)	-17.8 (16.6 – 19.0)	-0.020 (0.016 – 0.025)	
Liberia	DHS 2007	35.6		1,896
	DHS 2013	31.9		2,411
	% change (95% CI)	-10.4 (9.1 – 11.9)	-0.017 (0.012 – 0.024)	
Nigeria	DHS 2008	38.3		8,730
	DHS 2013	41.0		9,709
	% change (95% CI)	7.0 (6.5 – 7.6)	0.014 (0.012 – 0.017)	
Sierra Leone	DHS 2008	46.0		1,756
	DHS 2013	35.9		4,242
	% change	-22.0 (20.1 – 24.0)	-0.044 (0.035 – 0.055)	
Burkina Faso	DHS 2003	49.3		3,313
	DHS 2010	49.9		4,589
	% change	1.2 (0.9 – 1.6)	0.002 (0.001 – 0.004)	
Guinea	DHS 2005	60.0		1,741
	DHS 2012	50.3		2,505
	% change	-16.2 (14.5 – 18.0)	-0.023 (0.016 – 0.031)	
Mali	DHS 2006	70.6		3,814
	DHS 2012	59.0		2,647
	% change	-16.4 (15.2 – 17.6)	-0.027 (0.022 – 0.033)	
Niger	DHS 2006	74.1		2,306
	DHS 2012	76.3		2,698
	% change	3.0 (2.3 – 3.8)	0.005 (0.003 – 0.009)	
West Africa	Survey 1	43.5		
	Survey 2	41.5		40,201
	% change (95% C.I)	-4.6 (4.4 – 4.8)	-0.008 (0.007 – 0.009)	

Note: positive percentage changes indicate an increase while negative values indicate a decrease. The 95% confidence intervals (CI) are for absolute values of the percentage change. Women who were sexually inactive at the time of the surveys were excluded from the mean computation. DHS=Demographic and Health Surveys program.

### Correlates of child marriage

Rural residence and non-Christian religious status were significantly associated with higher prevalence of child marriage in each country in the Chi-square analysis of the most recent DHS (but the religious status was not available for Niger) while education and wealth quintiles had significant negative associations ( Table 3).

**Table 3.** Bivariate analysis of the association between selected socio-demographic factors and girl-child marriage in 11 West African countries using the most recent country-specific Demographic and Health Surveys (DHS) data.

					Percentage of females aged 18–24 years who were married before age 18						
	Ghana	Benin	Cote d’Ivoire	Senegal	Liberia	Nigeria	Sierra Leone	Burkina Faso	Guinea	Mali	Niger
Background characteristics	% with child marriage	% with child marriage	% with child marriage	% with child marriage	% with child marriage	% with child marriage	% with child marriage	% with child marriage	% with child marriage	% with child marriage	% with child marriage
**Residence**											
Rural	24.3	39.1	46.5	45.6	43.7	56.1	47.7	61.2	65.9	66.4	85.6
Urban	13.6	20.0	21.3	19.6	25.6	19.6	20.2	25.6	29.9	40.3	40.7
Chi-Square	24.60 ***	97.04 ***	54.56 ***	31.32 ***	38.28 ***	218.06 ***	87.49 ***	224.71 ***	104.13 ***	97.31 ***	274.48 ***
**Education**											
None	45.2	45.9	45.5	53.2	54.8	80.9	57.2	62.0	69.0	70.8	85.2
Primary	33.5	28.1	32.7	31.9	36.9	55.9	52.2	38.0	52.5	57.6	69.9
Secondary/higher	12.3	9.0	9.6	9.0	20.2	14.2	16.5	15.8	18.6	27.9	30.5
Chi-Square	76.83 ***	218.90 ***	88.42 ***	87.88 ***	45.22 ***	857.12 ***	215.21 ***	170.51 ***	148.60 ***	115.36 ***	188.15 ***
**Religion**											
Christianity	16.6	23.9	22.4	6.5	29.9	16.4	25.1	37.5	49.4	42.4	NA
Non-Christians ^b^	27.7	38.6	40.6	32.3	44.1	62.4	39.5	55.4	50.4	59.7	NA
Chi-Square	15.40 ***	62.85 ***	50.96 ***	25.20 ***	13.68 **	496.17 ***	27.49 ***	61.53 ***	0.03	11.76 ***	NA
**Wealth quintile** ^h^											
Poorest	31.7	48.7	58.3	59.0	47.6	81.2	52.5	66.4	74.0	71.8	85.4
Poorer	23.5	45.0	42.4	45.6	45.7	63.2	51.5	64.0	69.5	68.2	85.5
Middle	19.0	34.8	30.7	32.9	33.0	36.5	45.1	59.9	61.1	71.9	85.7
Richer	13.7	22.8	31.1	17.6	28.5	24.7	31.1	52.2	44.7	58.1	84.8
Richest	10.2	13.3	14.5	14.0	15.1	10.3	16.9	25.9	23.2	36.6	49.2
Chi-Square	11.05 ***	67.98 ***	29.60 ***	26.22 ***	16.89 ***	263.35 ***	47.49 ***	71.73 ***	60.56 ***	39.29 ***	58.96 ***
**Total**											

*Note:* respondents who married before age 18 were categorized as child marriage while those who were still unmarried or married at age 18 or above at the time of the survey were categorized as others. ^h^household wealth quintile; ^b^Non-Christians include Muslim, traditionalist, animist, no religion; NA data not available *** p < 0.001; ** p < 0.01; * p < 0.05

The logistic regression model including only residence, wealth quintile, and religion ( Table 4) shows that rural women had significantly higher odds for child marriage compared to urban women in all countries except Mali and the significant level was borderline for Ghana. For the nine countries with statistically significant findings, the adjusted odds ratio (AOR) ranged from 1.28 for Benin (95% confidence interval [CI]=1.05-1.57) to 5.60 for Niger (95% CI=3.78-8.30). Women of non-Christian religion had significantly higher odds for child marriage compared to Christian women in the ten countries with relevant data except Guinea: the odds were highest in Nigeria (AOR=5.86, 95% CI=4.96-6.92) and Senegal (AOR=7.25, 95% CI=3.18-16.54). Compared to the richest group, the three lowest economic categories had significantly higher odds in all the countries and the richer category had significantly higher odds too in all the countries except Ghana and Senegal.

**Table 4.** Multivariable logistics analysis of the association between selected socio-demographic variables (excluding education), and girl-child marriage in 11 West African countries using the most recent country-specific Demographic and Health Surveys (DHS) data.

	Ghana	Benin	Cote d’Ivoire	Senegal	Liberia	Nigeria	Sierra Leone	Burkina Faso	Guinea	Mali	Niger
Background Characteristics	AOR (95% CI)	AOR (95% CI)	AOR (95% CI)	AOR (95% CI)	AOR (95% CI)	AOR (95% CI)	AOR (95% CI)	AOR (95% CI)	AOR (95% CI)	AOR (95% CI)	AOR (95% CI)
**Residence**											
Urban ^RC^	1.00	1.00	1.00	1.00	1.00	1.00	1.00	1.00	1.00	1.00	1.00
Rural	1.37 (0.98–1.93)	1.28 * (1.05–1.57)	1.79 * (1.10–2.92)	1.38 (0.94–2.03)	1.20 (0.92–1.56)	2.11 *** (1.74–2.56)	1.74 ** (1.28–2.37)	2.46 *** (1.93–3.13)	1.63 * (1.11–2.40)	1.24 (0.86–1.80)	5.60 *** (3.78–8.30)
**Wealth quintile** ^h^											
Richest ^RC^	1.00	1.00	1.00	1.00	1.00	1.00	1.00	1.00	1.00	1.00	1.00
Richer	1.24 (0.66–2.31)	1.73 *** (1.31–2.82)	2.28 ** (1.42–3.65)	1.27 (0.66–2.42)	2.39 ** (1.31–4.34)	2.07 *** (1.61–2.66)	1.64 ** (1.16–2.32)	1.67 *** (1.26–2.22)	2.26 *** (1.62–3.16)	2.14 *** (1.51–3.06)	2.02 *** (1.39–2.94)
Middle	1.74 * (1.01–3.02)	2.74 *** (2.04–3.70)	1.94 ** (1.19 –3.15)	2.62 *** (1.62–4.22)	2.72 *** (1.70–4.37)	3.08 *** (2.37–4.01)	2.35 *** (1.57–3.52)	1.99 *** (1.50–2.65)	3.39 *** (2.19–5.26)	3.75 *** (2.45–5.74)	1.77 * (1.11–2.80)
Poorer	1.96 * (1.05–3.67)	4.02 *** (2.99–5.39)	2.59 ** (1.38–4.89)	4.05 *** (2.41–6.79)	4.35 *** (2.56–7.39)	6.67 *** (5.10–8.72)	2.97 *** (1.91–4.63)	2.40 *** (1.79–3.22)	4.79 *** (2.97–7.71)	3.19 *** (2.03–5.02)	1.68 * (1.06–2.64)
Poorest	2.72 ** (1.44–5.15)	4.51 *** (3.28–6.20)	4.77 *** (2.59–8.80)	6.88 *** (4.12–11.50)	4.53 *** (2.67–7.69)	10.55 *** (7.71–14.45)	3.11 *** (2.04–4.75)	2.58 *** (1.84–3.61)	5.84 *** (3.54–9.65)	3.86 *** (2.38–6.26)	1.64 * (1.02–2.65)
**Religion**											
Christianity ^RC^	1.00	1.00	1.00	1.00	1.00	1.00	1.00	1.00	1.00	1.00	
Non–Christians ^m^	1.70 ** (1.22–2.37)	1.52 *** (1.28–1.80)	2.50 *** (1.98–3.18)	7.25 *** (3.18–16.54)	1.96 *** (1.43–2.68)	5.86 *** (4.96–6.92)	1.69 *** (1.34–2.12)	1.84 *** (1.54–2.20)	1.13 (0.70–1.81)	2.40 *** (1.56–3.71)	NA

^RC^ Reference category; ^h^household wealth quintile; ^m^Muslim and others including traditionalist, animist, no religion; AOR adjusted odds ratio; NA data not available, CI confidence interval, *** p < 0.001; ** p < 0.01; * p < 0.05

In the second regression model ( Table 5), where education was added, education showed a significant negative association with girl-child marriage in all the 11 countries. Women with primary education had about 2–4 times higher odds of experiencing girl-child marriage compared to those with secondary/higher education, while those with no formal education had about 3–8 times higher odds. The pattern of association between child marriage and the other socio-demographic factors (residence, religion, and wealth quintiles) remains essentially the same as in the first regression model, although the AOR reduced slightly for each variable and some of the borderline associations in model 1 became non-significant in model 2.

**Table 5.** Multivariable logistics analysis of the association between selected socio-demographic variables (including education) and girl-child marriage in 11 West African countries using the most recent country-specific Demographic and Health Surveys (DHS) data.

	Ghana	Benin	Cote d’Ivoire	Senegal	Liberia	Nigeria	Sierra Leone	Burkina Faso	Guinea	Mali	Niger
Background Characteristics	AOR (95% CI)	AOR (95% CI)	AOR (95% CI)	AOR (95% CI)	AOR (95% CI)	AOR (95% CI)	AOR (95% CI)	AOR (95% CI)	AOR (95% CI)	AOR (95% CI)	AOR (95% CI)
**Residence**											
Urban ^RC^	1.00	1.00	1.00	1.00	1.00	1.00	1.00	1.00	1.00	1.00	1.00
Rural	1.32 (0.90–1.92)	1.18 (0.97–1.44)	1.52 (0.93–2.50)	0.94 (0.64–1.39)	1.15 (0.87–1.52)	1.86 *** (1.54–2.25)	1.40 * (1.06–1.86)	1.99 *** (1.55–2.54)	1.40 (0.96–2.03)	1.15 (0.81–1.63)	4.23 *** (2.90–6.17)
**Education**											
Secondary/higher	1.00	1.00	1.00	1.00	1.00	1.00	1.00	1.00	1.00	1.00	1.00
Primary	3.00 *** (2.19–4.11)	3.37 *** (2.59–4.39)	3.01 *** (2.10–4.32)	4.12 *** (2.89–5.88)	1.60 ** (1.14–2.23)	4.47 *** (3.73–5.35)	4.16 *** (3.29–5.26)	2.43 *** (1.80–3.28)	3.67 *** (2.70–4.97)	2.78 *** (1.94–3.96)	3.63 *** (2.54–5.19)
No education ^RC^	4.62 *** (3.20–6.69)	5.71 *** (4.51–7.23)	3.91 *** (2.83–5.39)	7.69 *** (2.89–5.88)	3.13 *** (2.35–4.17)	6.76 *** (5.52–8.27)	4.72 *** (3.84–5.81)	4.23 *** (3.14–5.71)	5.81 *** (4.25–7.94)	4.43 *** (3.37–5.83)	6.18 *** (4.43–8.62)
**Wealth quintile** ^h^											
Richest ^RC^	1.00	1.00	1.00	1.00	1.00	1.00	1.00	1.00	1.00	1.00	1.00
Richer	1.19 (0.64–2.22)	1.30 (0.97–1.74)	1.97 ** (1.21–3.19)	1.12 (0.56–2.22)	2.09 * (1.14–3.86)	1.58 *** (1.24–2.00)	1.38 (0.96–1.98)	1.26 (0.94–1.69)	1.80 ** (1.29–2.52)	1.48 * (1.06–2.07)	1.52 * (1.05–2.19)
Middle	1.53 (0.89–2.64)	1.78 *** (1.30–2.44)	1.53 (0.93 –2.51)	2.39 *** (1.50–3.82)	2.22 ** (1.35–3.64)	1.56 ** (1.20–2.03)	1.80 ** (1.21–2.66)	1.41 * (1.05–1.88)	2.21 ** (1.41–3.47)	2.16 *** (1.45–3.22)	1.26 (0.81–1.97)
Poorer	1.41 (0.74–2.70)	2.27 *** (1.66–3.09)	2.00 * (1.05–3.79)	3.17 *** (1.94–5.18)	3.18 *** (1.81–5.57)	2.42 *** (1.84–3.19)	2.03 ** (1.32–3.13)	1.64 ** (1.21–2.22)	2.59 *** (1.60–4.21)	1.62 * (1.07–2.46)	1.17 (0.75–1.82)
Poorest	1.55 (0.79–3.05)	2.25 *** (1.60–3.16)	3.46 *** (1.85–6.44)	5.35 *** (3.28–8.73)	2.83 *** (1.60–5.02)	3.03 *** (2.20–4.17)	1.83 ** (1.20–2.79)	1.73 ** (1.23–2.44)	2.94 *** (1.76–4.93)	1.95 ** (1.24–3.08)	1.07 (0.66–1.72)
**Religion**											
Christianity ^RC^	1.00	1.00	1.00	1.00	1.00	1.00	1.00	1.00	1.00	1.00	
Non–Christians ^m^	1.19 (0.84–1.68)	1.33 ** (1.12–1.57)	1.92 *** (1.50–2.46)	4.00 ** (1.79–8.95)	1.63 ** (1.22–2.17)	2.95 *** (2.46–3.54)	1.40 ** (1.11–1.76)	1.54 *** (1.29–1.84)	0.85 (0.56–1.31)	2.22 *** (1.44–3.41)	NA

^RC^ Reference category; ^h^household wealth quintile; ^m^Muslim and others including traditionalist, animist, no religion; AOR adjusted odds ratio; NA data not available, *** p < 0.001; ** p < 0.01; * p < 0.05F

## Discussion

Up-to-date data on the trends in child marriage in West African countries are needed for monitoring progress particularly in the light of the 2030 SDG targets but recent studies are lacking. This study addresses the current evidence gap. As our findings show, more than a quarter of young women experienced child marriage in 10 of the 11 countries studied while the only exception, Ghana, also has a high prevalence of 18.7%. At least half of the young women (age 18-24 years) in four countries – Burkina Faso, Guinea, Mali, and Niger – experienced girl-child marriage. Avogo and Somefun, in their study of three West African countries using DHS, reported a child marriage prevalence of 25% for Burkina Faso, 28% for Nigeria, and 59.7% for Niger ^19^. These rates are understandably lower than our findings as their analysis focused on adolescents (15-18 years), some of whom, though younger than 18 at the time of the study, may still experience child marriage before age 18. Studying age 15–18 as against age 18–24 years as we did underestimates the prevalence of child marriage. With the prevalence in our study ranging from 19.6% in Cote d’Ivoire to 74.1% in Niger in the first survey, and from 18.7% in Ghana to 76.3 in Niger in the second survey, there is almost a four-fold difference in prevalence between the countries with the highest and lowest levels of girl-child marriage in West Africa, implying considerable variation in burden. The increase of about two-thirds in girl-child marriage in Cote d’Ivoire between the two surveys (2005-2012) is puzzling and may not be unconnected to the socio-economic challenges that bedeviled the country during the political crisis that engulfed it during the 2010-2011 post-election violence. We recorded an overall high prevalence rate of 41.5% for girl-child marriage in West Africa for the most recent DHS. This figure compares well with the 2017 report of the United Nations Children’s Fund (UNICEF) that four in ten young women across the combined sub-regions of West and Central Africa had childhood marriage ^13^. UNICEF’s analysis incorporated data from both Multiple Cluster Indicator Surveys (for 6 of the countries covered in our study) and DHS (for the other 5 countries) conducted between 2010 and 2017 and covered 23 of the 24 West and Central African countries but did not disaggregate the result for the two sub-regions ^20^.

We found that the overall rate of progress in the reduction of girl-child marriage in West Africa is very slow, with a 4.6% reduction (from 43.5% to 41.5%) over approximately seven years and an annual percentage reduction of 0.01%. This reflects a similar trend to UNICEF’s report that child marriage decreased by a fifth in West and Central Africa over a 25-year period – from 50% in 1990 to 39% in 2015 ^13^. At the present rate, West African is not on course to meet the SDG goal. As UNICEF noted, it will take West and Central Africa about 100 years to eliminate girl-child at its current rate of progress ^21^. In addition, given the high population growth rate, West Africa will record an ever-higher number of girl-brides if the current low rate of progress continues. While substantial inter-country variations exist in child marriage trends across West Africa, none of the countries is on target for the SDG: Sierra Leone has the highest annual reduction rate (21% between 2008 and 2013 and an annual rate of 0.44 reduction in prevalence). Globally, the average annual rate of reduction in the prevalence of child marriage is 1.9% over the last 10 years whereas 23% reduction is required to meet the SDG target.

Rather than a decrease, three countries – Cote d’Ivore, Niger, and Nigeria – recorded an increase in the prevalence of girl-child marriage between the last two DHS while the rate remains about the same in Burkina Faso. Cote d’Ivore recorded more than a 50% increase (from 19.6% to 32.8%), while Niger, which has the highest rate of girl-child marriage globally ^7, 13^, had 3% increase (from 74.1% to 76.3%) and Nigeria, the most populous country in sub-Saharan Africa, had a 7% increase (from 38.3% to 41.0%). These findings imply the need for more vigorous efforts in West Africa to decrease the prevalence of child marriage. Clearly, without a significant acceleration in the rate of child marriage reduction in West Africa, the world is in no position to achieve the SDG given the high prevalence in the sub-region and its rate of progress that ranks as one of the slowest in the world ^9^. Moving forward, there is a need to examine the scope and scale of on-going interventions in each country. On the other hand, ongoing interventions need to be critically examined in terms of the potentials for impact as previous reviews showed that most programmes targeted at addressing child marriage in low-and-middle income countries were weak in design, lacked rigorous evaluation mechanisms, and low possibility of effectiveness ^22^.

In terms of determinants, in virtually all the countries, young women who live in rural settings, have lower education, and from poor households are at greater risk of becoming child-brides. Incidentally, almost three-fifths (56.9%) of young women across West Africa, as reflected from our study with the probability sampling approach, are rural-based, more than two-fifths (43.1%) have no formal education, and a third are from the poorer/poorest families (33.3%): these vulnerable populations need to be specially targeted with interventions that would effectively respond to their context and circumstances. Rural location, low education, and low household economic status as risk factors for child marriage are intricately connected and are different faces of poverty in the West African context. The rural population has limited economic and development opportunities, poorer access to quality schools, and lower access to sexual and reproductive health information and healthcare compared to the urban-based population. Taken together, these factors reflect a low state of human development and poverty as structural determinants of child marriage in West Africa ^23– 25^.

As evidenced in the literature, poor families, particularly those in rural areas, may give out their daughter for marriage early as an economic “survival” measure to ease financial difficulty through a twin mechanism of receiving dowry or bride price and eliminating their direct expenditure on the girl ^7, 12, 14^. In settings where school fees are required, financial difficulties may cause poor families to withdraw their daughters from school, thereby increasing her risk for child marriage as schooling projects the perspective of the girl still being a child rather than a bride-in-waiting. In our study, lack of formal education was associated with a 3–8 increase in the odds of girl-child marriage while the attainment of only primary school education had 2–4 increased odds. Early cessation of schooling also disrupts the vital process of acquiring the human capital needed by adolescent girls and young women to break free from poverty in the future ^20^. In terms of religious affiliation, we found the odds of child marriage to be higher among non-Christians compared to Christians. While this result agrees with some published works that have reported a higher rate of child marriage among Muslims compared to other groups ^19, 26, 27^, this relationship does not lend itself to a simplistic interpretation, particularly as the influence of religion may not be easily separatable from that of the background sociocultural factor in the West African context. In addition, religion as an explanatory variable may be tied to or simply mask the political-economic factors, particularly in the West African countries with high Muslim population.

Our data did not include variables on patriarchy which may be regarded as an unexplored factor in our analysis. Previous research has established the influence of the patriarchal system in West Africa and its associated gender-inequitable norms, which become accentuated as the girl approaches puberty ^28, 29^ and plays a key role in girl-child marriage practice in the sub-region as it provides the context whereby the girl-child is less valued compared to the boy-child. As the patriarchy theory posits, patriarchal societies are characterized by the domination of males over females in almost all aspects of life, and unequal power relations pervade the health, economic, social and political landscapes ^30^. In the case where a poor family has to withdraw a child from school on account of unaffordable school fees, for example, the girl-child would likely be withdrawn. A key limitation of our study is that our data are based on self-report. However, with no social pressure against early marriages in many West African traditional settings, there may be little or no concern regarding the validity of self-report findings. Another limitation is that our study is cross-sectional in design and, therefore, no causality can be deduced for any reported associations.

Overall, our findings indicate that the rate of girl-child marriage remains very high in West Africa, but very little progress is being recorded, at present, towards ending child marriage in the sub-region. In addition, substantial inter-country variation exists in the overall level of child marriage as well as the rate of progress, but no country is on target to achieve the SDG given the current low rate of progress. There is, therefore, the need to develop and implement effective actions against girl-child marriage in all West African countries to rapidly accelerate progress towards ending child marriage. With the roles of the socio-demographic factors highlighted in this study, efforts to reduce girl-child marriage in West Africa need to address the social and economic structures that underlie the practice to achieve effective and sustainable results. Engineering structural change in a society may be an uphill task, but it is important to address structural constraints that serve as impediments to reducing child marriage across West Africa. The determinants highlighted in the study have utility for designing appropriate interventions and also for prioritising target populations for intervention in the resource-challenged settings of West Africa.

## Data availability

Data are from the DHS Program and are publicly available via https://dhsprogram.com/data/. To access the data, readers must register as a DHS data user via: https://dhsprogram.com/data/new-user-registration.cfm.
