# Supplementary material for: Trends and correlates of girl-child marriage in 11 West African countries: evidence from recent Demographic and Health Surveys
Source: AAS Open Res. 2021 Sep 1;4:35. Originally published 2021 Jun 22. [Version 2] doi: 10.12688/aasopenres.13248.2 (PMC8431189; doi:10.12688/aasopenres.13248.2)
Supplement: Peer review history

# Peer review: Reviewer response for version 2

**DOI:** 10.21956/aasopenres.14417.r28862

**Published:** 2022-01-04

**Authors:** Dieudonné Uwizeye

**Affiliations:** 1 Department of Development Studies, University of Rwanda, Kigali, Rwanda

The authors have adequately addressed all the comments. I have no further comments to make.

Is the work clearly and accurately presented and does it cite the current literature?

Yes

If applicable, is the statistical analysis and its interpretation appropriate?

Yes

Are all the source data underlying the results available to ensure full reproducibility?

Yes

Is the study design appropriate and is the work technically sound?

Yes

Are the conclusions drawn adequately supported by the results?

Yes

Are sufficient details of methods and analysis provided to allow replication by others?

Yes

Reviewer Expertise:

Demography and population studies; population and development; research capacity strengthening; survey designs and community-based study designs.

I confirm that I have read this submission and believe that I have an appropriate level of expertise to confirm that it is of an acceptable scientific standard.

# Peer review: Reviewer response for version 2

**DOI:** 10.21956/aasopenres.14417.r28861

**Published:** 2021-09-09

**Authors:** Eunice N. S. Imasiku

**Affiliations:** 1 Department of Geography and Environmental Studies, School of Natural Sciences, University of Zambia, Lusaka, Zambia

I have gone through the revised version of the article by Fatusi, Adedini and Mobolaji and hereby confirm that the authors have adequately attended to all the concerns raised in my previous report. Therefore, my status of 'Approved with reservations' can be changed to 'Approved'.

Is the work clearly and accurately presented and does it cite the current literature?

Yes

If applicable, is the statistical analysis and its interpretation appropriate?

Yes

Are all the source data underlying the results available to ensure full reproducibility?

Yes

Is the study design appropriate and is the work technically sound?

Yes

Are the conclusions drawn adequately supported by the results?

Yes

Are sufficient details of methods and analysis provided to allow replication by others?

Yes

Reviewer Expertise:

NA

I confirm that I have read this submission and believe that I have an appropriate level of expertise to confirm that it is of an acceptable scientific standard.

# Peer review: Reviewer response for version 1

**DOI:** 10.21956/aasopenres.14370.r28721

**Published:** 2021-08-03

**Authors:** Eunice N. S. Imasiku

**Affiliations:** 1 Department of Geography and Environmental Studies, School of Natural Sciences, University of Zambia, Lusaka, Zambia

The main objective of the paper was to determine the prevalence and compare trends of child marriage in West Africa. Moreover, it also assessed the relationship between child marriage and selected socio-demographic factors. The paper has potential to make valuable contribution to literature on child marriage as well as giving us a picture on how West African Countries are faring in meeting SDG target 5.3. The paper is well written and contains the components expected to be in such a paper.

 However, I have the following observations:

**Introduction:**

 Since the paper has no section for literature review, the authors should consider including something from existing literature to put the topic in context especially on the factors associated with child marriage. Though in the methods section the authors state that the explanatory variables included in the analysis were based on existing literature, this is not evident in the paper.

**Methods:**

 It is not clear why the wealth variable was categorized as poorest, poorer, middle, richer and richest in some instances and poorest/poorer, middle and richer/richest in other instances.

**Results:**

*Trends in child marriage:*

 Putting Table 2 at the end of the first sentences in this sub-section suggests that only what is in that sentence is in table 2 and as such what follows is not associated with that table. It is not clear what is meant by Burkina Faso’s rate being virtually unchanged.

*Correlates of child marriage*

 In the second paragraph under this sub-section, the authors refer to nine countries having statistically significant findings and yet they are seven according to Table 4. The authors can consider having three categories for wealth instead of five. The last paragraph indicates that secondary/higher education is the reference category but Table 5 indicates that no education is the reference category.

**Discussion:**

 The authors can also consider comparing their findings with those of other sub-regions of Africa or other developing countries. It is not clear why the authors suddenly refer to the patriarchy theory in the discussion when this is not referred to before that. Actually the mentioning of their not including variables on patriarchy at the beginning of the paragraph dealing with this makes one wonder the relevance of that paragraph.

Is the work clearly and accurately presented and does it cite the current literature?

Yes

If applicable, is the statistical analysis and its interpretation appropriate?

Yes

Are all the source data underlying the results available to ensure full reproducibility?

Yes

Is the study design appropriate and is the work technically sound?

Yes

Are the conclusions drawn adequately supported by the results?

Yes

Are sufficient details of methods and analysis provided to allow replication by others?

Yes

Reviewer Expertise:

NA

I confirm that I have read this submission and believe that I have an appropriate level of expertise to confirm that it is of an acceptable scientific standard, however I have significant reservations, as outlined above.

# Response

**Published:** 2021-08-25

**Authors:** Sunday Sunday

**Affiliations:** Federal University Oye-Ekiti, Nigeria

**Reviewer 2: Dr Eunice Imasiku:**

**Comments:**

 The main objective of the paper was to determine the prevalence and compare trends of child marriage in West Africa. Moreover, it also assessed the relationship between child marriage and selected socio-demographic factors. The paper has potential to make valuable contribution to literature on child marriage as well as giving us a picture on how West African Countries are faring in meeting SDG target 5.3. The paper is well written and contains the components expected to be in such a paper.

**Response:**

 Thank you.

**Comments**

 However, I have the following observations:

 Introduction:

 Since the paper has no section for literature review, the authors should consider including something from existing literature to put the topic in context especially on the factors associated with child marriage. Though in the methods section the authors state that the explanatory variables included in the analysis were based on existing literature, this is not evident in the paper.

**Response:**

 A review of relevant literature has been included in the revised introduction, showing the factors associated with child marriage. All changes are tracked up. Some of the characteristics from the reviewed literature are presented below:

 Child marriage is an age-long practice influenced by poverty, low education, religious beliefs, traditional norms and cultural beliefs, inequitable gender norms and low social status of women, among others.

**Comments:**

 Methods:

 It is not clear why the wealth variable was categorized as poorest, poorer, middle, richer and richest in some instances and poorest/poorer, middle and richer/richest in other instances.

**Response:**

 In the descriptive analysis, we present three categories of wealth index basically to show the percentage distribution. We used the original five categories for the multivariable analysis. This explanation has been provided in the revised manuscript.

**Comments:**

 Results:

 Trends in child marriage:

 Putting Table 2 at the end of the first sentences in this sub-section suggests that only what is in that sentence is in table 2 and as such what follows is not associated with that table. It is not clear what is meant by Burkina Faso’s rate being virtually unchanged.

**Response:**

 We have moved the phrase ‘Table 2’ to the beginning of that sub-section. Also, we have revised the sentence on Burkina Faso to what is shown below:

 Burkina Faso’s rate remained unchanged over the study period.

**Comments:**

 Correlates of child marriage

 In the second paragraph under this sub-section, the authors refer to nine countries having statistically significant findings and yet they are seven according to Table 4. The authors can consider having three categories for wealth instead of five. The last paragraph indicates that secondary/higher education is the reference category but Table 5 indicates that no education is the reference category.

**Response:**

 We have corrected the number of countries with significant results to seven instead of nine. The appropriate number of categories for wealth index/wealth quintile is five and this is what was used in the multivariable analysis. ‘secondary/higher education’ is the correct reference category for education variable and we have corrected this accordingly.

                                           

**Comments:**

 Discussion:

 The authors can also consider comparing their findings with those of other sub-regions of Africa or other developing countries. It is not clear why the authors suddenly refer to the patriarchy theory in the discussion when this is not referred to before that. Actually the mentioning of their not including variables on patriarchy at the beginning of the paragraph dealing with this makes one wonder the relevance of that paragraph.

**Response:**

 Although the manuscript is focused on West Africa, the discussion has been revised to include some information on how our findings compare with those of other sub-regions. As suggested, we have removed patriarchy theory and explanation about patriarchy variable from the discussion section.

 Is the work clearly and accurately presented and does it cite the current literature?

 Yes

 Is the study design appropriate and is the work technically sound?

 Yes

 Are sufficient details of methods and analysis provided to allow replication by others?

 Yes

 If applicable, is the statistical analysis and its interpretation appropriate?

 Yes

 Are all the source data underlying the results available to ensure full reproducibility?

 Yes

 Are the conclusions drawn adequately supported by the results?

 Yes

**Response:**

 Thank you.

# Peer review: Reviewer response for version 1

**DOI:** 10.21956/aasopenres.14370.r28720

**Published:** 2021-08-02

**Authors:** Dieudonné Uwizeye

**Affiliations:** 1 Department of Development Studies, University of Rwanda, Kigali, Rwanda

It has been a great honour and a privilege to review the article entitled " *Trends and correlates of girl-child marriage in 11 West African countries: evidence from recent Demographic and Health Surveys*." I appreciate the authors for a well-written document and interesting topic. The paper articulated fairly deeply the issue of girl-child marriage in West Africa, and indicated that the place of residents, education levels, household economic status and religious affiliation “ *were significantly associated with higher odds of girl-child marriage*”.

 However, I have identified some comments which I believe the authors should review to improve the manuscript more:

**1) Typos, and grammatical errors:**

- **i) **In the “ Introduction” section, paragraph 2; last sentence: The concept of “ *girl-child marriage*” changed to “ *child-girl marriage*.” The authors may need to be consistent on how this concept is used to avoid confusion.
- **ii)** In the “ Introduction” section; paragraph 3; last sentence: the manuscript quoted the title of the Maputo Protocol but missed the last word of the title: “in Africa”. I think the authors may consider using the full title of the instrument. Here is the link to the correct title: https://au.int/en/treaties/protocol-african-charter-human-and-peoples-rights-rights-women-africa.
- **iii)** In the “ Discussion (page 9)”; paragraph 2; sentence 4: the sentence reads as if the word “ marriage” is missing:
  “ *As* *UNICEF noted, it will take West and Central Africa about 100 years to eliminate girl-child MARRIGE at its current rate of progress*”.
- **iv)** In the “ Discussion (page 11); paragraph 5, second sentence; there is a grammatical error: “…increasing her risk…” should read “…increasing their risk…”. " *In settings where school fees are required, financial difficulties may cause poor families to withdraw their daughters from school, thereby increasing her risk for child marriage as schooling projects the perspective of the girl still being a child rather than a bride-in-waiting*”.

 

**2) Content-based comments:**

**(i) Statements of the objective of the study**: The title of the paper indicates very well the objective of the study of using the DHS data from 11 countries to assess “the trends and correlates of girl-child marriage”. All the sections of the paper are articulated very well by this objective, and there is no problem with it. However, there are instances in the paper where the statement of the objective is a little bit confusing:

- In the abstract, the last part of the first sentence of the methods reads: *“…to determine the prevalence and trend of girl-child marriage.” *
- In the methods section, the last part of the 4 ^th^ sentence reads: *“… to assess trends in child marriage, and the most recent survey to determine the current prevalence and correlates.”*
- The authors may need to revisit these two sentences to ensure that there are no confusing statements of the objectives of the study.

**(ii) Data presentation and discussion: **

- **The second paragraph of the “introduction**” section opens with useful evidence of the burden of child marriage. However, it is not clear whether the statement refers to a global burden, or whether it is about West Africa, the setting of the paper or any other part of the world. The authors need to clearly position the evidence geographically.
- **In the discussion section**, specifically, paragraph 3, the authors may need to check if the passage is not redundantly presenting the data (rather than discussing) which were already presented in the results section.

Is the work clearly and accurately presented and does it cite the current literature?

Yes

If applicable, is the statistical analysis and its interpretation appropriate?

Yes

Are all the source data underlying the results available to ensure full reproducibility?

Yes

Is the study design appropriate and is the work technically sound?

Yes

Are the conclusions drawn adequately supported by the results?

Yes

Are sufficient details of methods and analysis provided to allow replication by others?

Yes

Reviewer Expertise:

Demography and population studies; population and development; research capacity strengthening; survey designs and community-based study designs.

I confirm that I have read this submission and believe that I have an appropriate level of expertise to confirm that it is of an acceptable scientific standard.

# Response

**Published:** 2021-08-25

**Authors:** Sunday Sunday

**Affiliations:** Federal University Oye-Ekiti, Nigeria

**Response to reviewers’ comments**

**Reviewer 1: Dr Dieudonné Uwizeye**

**Comments:**

 It has been a great honour and a privilege to review the article entitled "Trends and correlates of girl-child marriage in 11 West African countries: evidence from recent Demographic and Health Surveys." I appreciate the authors for a well-written document and interesting topic. The paper articulated fairly deeply the issue of girl-child marriage in West Africa, and indicated that the place of residence, education levels, household economic status and religious affiliation “were significantly associated with higher odds of girl-child marriage”.

**Response:**

 Thank you.

**Comments:**

 However, I have identified some comments which I believe the authors should review to improve the manuscript more:

 1) Typos, and grammatical errors:

 i) In the “Introduction” section, paragraph 2; last sentence: The concept of “girl-child marriage” changed to “child-girl marriage.” The authors may need to be consistent on how this concept is used to avoid confusion.

**Response:**

 For consistency’s sake, the same terminology “girl-child marriage” has been used throughout the manuscript.

**Comments:**

 ii) In the “Introduction” section; paragraph 3; last sentence: the manuscript quoted the title of the Maputo Protocol but missed the last word of the title: “in Africa”. I think the authors may consider using the full title of the instrument. Here is the link to the correct title: https://au.int/en/treaties/protocol-african-charter-human-and-peoples-rights-rights-women-africa.

**Response:**

 Thank you. We have quoted the correct title of the Maputo Protocol in the revised paper as shown below:

 Protocol to the African Charter on Human and Peoples' Rights on the Rights of Women in Africa

**Comments:**

 iii) In the “Discussion (page 9)”; paragraph 2; sentence 4: the sentence reads as if the word “marriage” is missing:

 “As UNICEF noted, it will take West and Central Africa about 100 years to eliminate girl-child MARRIGE at its current rate of progress”.

**Response:**

 The sentence has been corrected with the word “marriage” inserted into the appropriate place as shown in the text below:

 ‘As UNICEF noted, it will take West and Central Africa about 100 years to eliminate girl-child marriage at its current rate of progress.’

**Comments:**

 iv) In the “Discussion (page 11); paragraph 5, second sentence; there is a grammatical error: “…increasing her risk…” should read “…increasing their risk…”. "In settings where school fees are required, financial difficulties may cause poor families to withdraw their daughters from school, thereby increasing her risk for child marriage as schooling projects the perspective of the girl still being a child rather than a bride-in-waiting”.

**Response:**

 As pointed out, the grammatical error has been corrected in the revised paper. The correction is shown below:

 ‘In settings where school fees are required, financial difficulties may cause poor families to withdraw their daughters from school, thereby increasing their risk for child marriage ...’

**Comments:**

 2) Content-based comments:

 (i) Statements of the objective of the study: The title of the paper indicates very well the objective of the study using the DHS data from 11 countries to assess “the trends and correlates of girl-child marriage”. All the sections of the paper are articulated very well by this objective, and there is no problem with it. However, there are instances in the paper where the statement of the objective is a little bit confusing:

 In the abstract, the last part of the first sentence of the methods reads: “…to determine the prevalence and trend of girl-child marriage.”

 In the methods section, the last part of the 4th sentence reads: “…to assess trends in child marriage, and the most recent survey to determine the current prevalence and correlates.”

 The authors may need to revisit these two sentences to ensure that there are no confusing statements of the objectives of the study.

**Response:**

 We did examine the prevalence and trend of girl-child marriage as well as identified its correlates. Therefore, the sentences being referred to in the above comments clearly present the objectives of this study.

**Comments:**

 (ii) Data presentation and discussion:

 The second paragraph of the “introduction” section opens with useful evidence of the burden of child marriage. However, it is not clear whether the statement refers to a global burden, or whether it is about West Africa, the setting of the paper or any other part of the world. The authors need to clearly position the evidence geographically.

**Response:**

 The statement about child marriage refers to the global burden. This change has been effected in the revised paper.

**Comments:**

 In the discussion section, specifically, paragraph 3, the authors may need to check if the passage is not redundantly presenting the data (rather than discussing) which were already presented in the results section.

**Response:**

 We have expunged the result presentation being referred to in the discussion section.

**Comments:**

 Is the work clearly and accurately presented and does it cite the current literature?

 Yes

 Is the study design appropriate and is the work technically sound?

 Yes

 Are sufficient details of methods and analysis provided to allow replication by others?

 Yes

 If applicable, is the statistical analysis and its interpretation appropriate?

 Yes

 Are all the source data underlying the results available to ensure full reproducibility?

 Yes

 Are the conclusions drawn adequately supported by the results?

 Yes

**Response:**

 Thank you.
